# Analysis of molecular subtypes and antibiotic resistance in *Treponema pallidum* isolates from blood donors in Khyber Pakhtunkhwa, Pakistan

**DOI:** 10.1371/journal.pone.0305720

**Published:** 2024-06-21

**Authors:** Iqbal Muhammad, Eman H. Khalifa, Magdi M. Salih, Waheed Ullah, Manal S. A. Elseid, Muhammad Qasim, Sajid Ali, Nadeem Ullah, Noor Muhammad

**Affiliations:** 1 Department of Microbiology, Kohat University of Science and Technology, Kohat, Pakistan; 2 Department of Laboratory Medicine, Faculty of Applied Medical Sciences, University of Al Baha, Al Baha, Kingdom of Saudi Arabia; 3 Clinical Laboratory Sciences Department, College of Applied Medical Sciences, Taif University, Taif, Kingdom of Saudi Arabia; 4 Department of Medical Laboratory Sciences, Faculty of Applied Medical Sciences, Northern Border University, Arar, Kingdom of Saudi Arabia; 5 Department of Biotechnology, Bacha Khan University, Charsadda, Pakistan; 6 Department of Clinical Microbiology, Umeä University Hospital Umeä, Umeä, Sweden; Rutgers New Jersey Medical School, UNITED STATES

## Abstract

Syphilis, caused by *Treponema pallidum*, is resurging globally. Molecular typing allows for the investigation of its epidemiology. In Pakistan and other nations, *T. pallidum* subsp. *pallidum* has developed widespread macrolide resistance in the past decade. A study at the Peshawar Regional Blood Centre from June 2020–June 2021 analyzed serum samples from 32,812 blood donors in Khyber Pakhtunkhwa, Pakistan, to assess circulating *T. pallidum* strains and antibiotic resistance. Blood samples were initially screened for *T. pallidum* antibodies using a chemiluminescent microparticle immunoassay (CMIA). CMIA-reactive samples underwent polymerase chain reaction (PCR) targeted the *polA*, *tpp47*, *bmp*, and *tp0319* genes. PCR-positive samples were further analyzed for molecular subtyping using a CDC-developed procedure and *tp0548* gene examination. All PCR-positive samples were analyzed for the presence of point mutations A2058G and A2059G in 23S rRNA, as well as the G1058C mutation in 16S rRNA. These mutations are known to impart antimicrobial resistance to macrolides and doxycycline, respectively. Out of 32,812 serum samples, 272 (0.83%) were CMIA-reactive, with 46 being PCR-positive. Nine *T. pallidum* subtypes were identified, predominantly 14d/f. The A2058G mutation in 23S rRNA was found in 78% of cases, while G1058C in 16S rRNA and A2059G in 23S rRNA were absent. The research found donor blood useful for assessing *T. pallidum* molecular subtypes and antibiotic resistance, especially when chancres are not present. The prevalent subtype was 14d/f (51.85%), and the high macrolide resistance of 36 (78%) indicates caution in using macrolides for syphilis treatment in Khyber Pakhtunkhwa, Pakistan.

## 1. Introduction

*Treponema pallidum* subsp. *pallidum* causes the global sexually transmitted disease syphilis. In 2020, WHO reported 7 million new syphilis cases globally. Progress towards WHO’s 2030 goal to reduce syphilis by 90% is slow, with rising cases in some countries, particularly affecting pregnant women. Untreated or inadequately treated maternal syphilis can lead to adverse birth outcomes in 50–80% of cases [1]. In 2019, the United States recorded 129,813 syphilis cases [2]. In 2021, the syphilis infection rate in the USA was 53.0 cases per 100,000 people [3]. From 2010 to 2015, syphilis cases in Canada increased from 5.0 to 9.3 per 100,000 people [4]. In 2019, 29 EU/EEA countries recorded 35,039 syphilis cases with a rate of 7.4 cases per 100,000. Men, particularly those aged 25 to 34, had rates nine times higher than women, reaching 31 cases per 100,000 [5]. Syphilis rates among pregnant women vary regionally: 6.5% in Southern Africa, 4.6% in East Africa, and 4.0% in West Africa [6]. In 2020, Iran had a 0.1% syphilis prevalence, encompassing primary and secondary cases [7]. In 2019, syphilis diagnosis rate in South China was 112 per 100,000 for older individuals and 48.1 per 100,000 for younger individuals, indicating a significant age-related difference [8]. In global studies, the 14d/f subtype of *T. pallidum* is consistently found as the most common, observed in regions like the North America [9, 10], South America [11, 12], South Africa [13], Europe [14], Asia [9, 15–17]. In other studies, where macrolide resistance was identified, North America, (28.6%) [18], (68%) [19], (53.2%) [20], (16%) [21], (72%) [10], (64.34%) [22], South America, (14.3%) [11], (0%) [12], Australia, (84%) [23], Europe, (66.6%) [24], (100%) [25], (94.3%) [26], (93.1%) [27], (66.7%) [28], South Africa(23%) [13], Asia, (97.5%) [15], (91.9%) [29], (100%) [30]. Limited data in Pakistan hinders clarity on syphilis prevalence, particularly regarding molecular typing of *T. pallidum* and drug resistance. A 2018 nationwide study found 0.72% of blood donors tested positive for syphilis [31]. Syphilis is prevalent among blood donors in diverse areas of Pakistan, as indicated by multiple studies, 3.1% [32], 1.55% [33], 2.1% [34], and 0.91% [35]. From June 2016 to May 2020, Peshawar blood donors had a 0.91% syphilis rate [36]. Khyber Pakhtunkhwa had a 0.83% syphilis rate from June 2020-June 2021 [37].

In 1998, CDC researchers developed a molecular strain typing method to study pathogen diversity and disease epidemiology, specifically targeting *T. pallidum*, the syphilis-causing agent [38]. The CDC typing method and sequence analysis of *tp0548* gene segment are widely used for subtyping *T. pallidum* [9]. Over the last decade, molecular subtyping of *T. pallidum* has emerged to enhance local monitoring and managing of syphilis epidemics [9, 38]. This method improves syphilis comprehension, aids in identifying reinfection and relapse, and distinguishes between strains linked to particular symptoms [9]. Early syphilis chancres may self-heal unnoticed. Identifying the syphilis pathogen, *T. pallidum*, is impractical in clinical settings, such as dark field microscopy. Bacterial burden peaks in the secondary stage [39]. Collecting samples from chancres is more challenging than obtaining blood samples. The process relies on the accessibility of suitable health services for individuals with sexually transmitted infections to ensure sample collection and diagnosis. Blood samples are preferred for PCR diagnosis, resistance monitoring, and molecular subtyping of syphilis. While benzathine penicillin G is the standard treatment, the primary concern with benzathine penicillin G treatment is the availability issue and the sensitivity of some individuals to beta-lactam antibiotics, rather than drug resistance but antibiotic resistance can cause treatment failures. Azithromycin and doxycycline are alternative medications for patients allergic to penicillin or those who reject injections [40]. Macrolide resistance is widespread in China, the US, Canada, Australia, and Europe [10, 18–20, 23, 24, 27, 29], and it is connected to the 23S rRNA gene of *T. pallidum’s* A2058G and A2059G mutations [41, 42]. In 2014 European guidelines, doxycycline was recommended as an alternative treatment for latent syphilis when penicillin was not possible [40]. Research is required to examine G1058C mutations in spirochetes, particularly in Pakistani regions such as Khyber Pakhtunkhwa, where data on tetracycline resistance is lacking.

The aim of this research was to examine blood samples obtained from 272 blood donors in Khyber Pakhtunkhwa, Pakistan, who were previously identified as reactive for syphilis during our analysis conducted between June 2020 and June 2021 [37]. The goal was to investigate the molecular epidemiology of syphilis and identify point mutations associated with resistance to doxycycline and macrolides.

## 2. Materials and methods

This research, conducted on the samples we had previously analysed from June 2020 to June 2021 [37], involved a prospective, cross-sectional investigation at the Peshawar Regional Blood Centre. The Institutional Ethics Committees of the Regional Blood Centre in Peshawar, Khyber Pakhtunkhwa, Pakistan, and Kohat University of Science and Technology in Kohat, Pakistan, approved this study. This centre is responsible for collecting blood donations from individuals in the Peshawar region and distributing prepared blood components to hospitals throughout Peshawar, which serves a population of 4.7 million people. Additionally, due to its proximity to the Afghan border, the centre also provides blood components to a substantial number of Afghan refugees. Khyber Pakhtunkhwa (KP) is a province located in the north-western part of Pakistan, comprising 37 districts with a total population of 40.8 million. Peshawar, as the provincial capital and a well-developed city with advanced healthcare services, attracts residents from other districts who frequently seek superior healthcare. The blood donors included in this study were from various regions within KP, with a significant representation of Afghan ethnicities.

### 2.1. Clinical specimens

In this study, 5 mL of blood was extracted from each blood donor using plain tubes, specifically red-top tubes. All blood samples were collected with the explicit, informed consent of the donors. The serum from these red-top tubes was used to screen blood donors for syphilis using the CMIA technique [43]. A total of 32,812 samples were subjected to this testing method. The CMIA was employed to detect antibodies against *T. pallidum* in the serum of the blood donors. Subsequently, all serum samples that tested reactive for *T. pallidum* using CMIA underwent additional molecular analysis.

### 2.2. DNA extraction from CMIA reactive serum samples by spin protocol

According to the protocols recommended by the manufacturer within 24 hours of collection, the QIAamp DNA Blood Mini Kit (QIAGEN) was used for the extraction of DNA from all CMIA reactive samples for antibodies to *T. pallidum*. For the extraction of DNA, 200 μl of serum was used. The extracted DNA was preserved in Buffer AE and stored in a freezer at −20°C before further analysis [9].

### 2.3. Detection of *T. pallidum* DNA by PCR

Before subtyping and analysis for the discovery of markers specific for antibiotic resistance, all *T. pallidum*-reactive samples identified by CMIA were submitted to PCR using primers specific for the *polA*, *tpp47*, *bmp*, and *tp0319* genes of *T. pallidum*. To avoid contamination, distinct rooms were utilized for reagent preparation, DNA extraction, sample amplification, and subsequent PCR product analysis. In this study, the positive control included DNA derived from *T. pallidum* SS14 and Nichols strains, provided by the US-CDC, while distilled water was utilised as the negative control. PCR experiments were carried out in a volume of 50 μL, with 5 μL of DNA template obtained from the test subjects’ clinical materials or the controls [18]. A 2.0% agarose gel was used to identify the amplicons of the PCR. The gels were operated at 100 V of current for one hour with a 100-bp ladder. Following staining with ethidium bromide, bands were visible on a UV transilluminator [44]. Samples that tested PCR-positive for any of the specified genes were considered to contain *T. pallidum* DNA, and these samples were subsequently used to assess the molecular subtype of the DNA and its resistance to antibiotics. The primers and their sequences used to identify *T. pallidum* DNA by PCR are listed in Table 1.

**Table 1:** Primers employed in the molecular examination of *T. pallidum*.

Types of Analysis	Target Gene	Forward Primer	Reverse Primer
Screening	*tp0319*	F1: 5’-GAAGGTGGTGACTTCGTCGT-3’	R1: 5’-CAAAACCCGCTTCAAAGAGA-3’
	*polA*	F2: 5’-TGCGCGTGTGCGAATGGTGTGGTC-3’	R2: 5’-CACAGTGCTCAAAAACGCCTGCACG-3’
	*tpp47*	F3: 5’-TTGTGGTAGACACGGTGGGTAC-3’	R3: 5’-TGATCGCTGACAAGCTTAGGCT-3’
	*bmp*	F4: 5’-CAGGTAACGGATGCTGAAGT-3’	R4: 5-CGTGGCAGTAACCGCAGTCT-3’
Subtyping	*arp*	F5: 5’-ATCTTTGCCGTCCCGTGTGC-3’	R5: 5’-CCGAGTGGGATGGCTGCTTC-3
	*tprEGJ*	F6: 5’-ACTGGCTCTGCCACACTTGA-3’ F7: 5’-CAGGTTTTGCCGTTAAGC-3’	R6: 5’-CTACCAGGAGAGGGTGACGC-3’ R7: 5’-AATCAAGGGAGAATACCGTC-3’
	*tp0548*	F8: 5’-GGTCCCTATGATATCGTGTTCG-3’	R8: 5’-GTCATGGATCTGCGAGTGG-3’
Mutation	23S rRNA	F9: 5’-GTACCGCAAACCGACACAG-3’	R9: 5’-AGTCAAACCGCCCACCTAC-3’
	16S rRNA	F10: 5’-GTGGATGAGGAAGGTCGAAA-3’ F11: 5’-TCAACTTGGGAACTGCACTG-3’	R10: 5’-CAGAGTCCCCAACACCACTT-3’

### 2.4. Molecular subtyping of *T. pallidum*

Molecular subtyping was conducted on all PCR-positive specimens, and the specific primers used for this procedure are listed in Table 1. The molecular categorization of *T. pallidum* was achieved by combining three different analyses, which included the *arp* gene, *tpr* EGJ genes, and *tp0548* gene [9, 19, 45].

#### 2.4.1. Determining the number of 60-base pair repeats in the *arp* gene

A particular primer was used in PCR to count the number of 60-bp repeats in the *arp* gene. The results of the PCR were separated using agarose gel electrophoresis. The size of each PCR product was determined using the Quantity One software (version 4.6.7; Biorad), which was compared to a 1-kb DNA ladder (Invitrogen), and the number of 60-bp repeats in the *arp* gene was determined by comparing the size of the amplified PCR product to the PCR product amplified from the Nichols strain of *T. pallidum*, which carries 14 repeats [38].

#### 2.4.2. Performing a restriction fragment length polymorphism (RFLP) analysis of the *tpr* subfamily II (*tpr* EGJ) genes

To amplify the *tpr* genes, nested PCR was used. The previously mentioned amplification procedure in Section 2.3 and the two primer pairs were applied [44]. The *tpr* EGJ genes were amplified using an outer primer pair. Internal primers were used in the second round of PCR, using the first round’s amplicon as a template. The nested PCR product was purified using a QIAquick PCR Purification Kit from Qiagen, digested with a quick restriction endonuclease, and then electrophoresed on a 2.0% agarose gel to determine the digestion pattern through comparison with previously published data [38].

#### 2.4.3. Examining the variable regions in the *tp0548* gene, as previously documented

For the examination of the *tp0548* gene’s variable region from positive samples, the previously published primer set was utilized to amplify the variable region of the *tp0548* gene. Using a QIAquick PCR Purification Kit from Qiagen, PCR products were purified before being sequenced for genotyping. The reference sequences provided by Marra *et al*. were matched to the sequence type of *tp0548* [9].

### 2.5. Characterization of drug resistance genes in *T. pallidum*

#### 2.5.1. Detection of the 23S rRNA A2058G and A2059G mutations

The point mutations A2058G and A2059G within the 23S rRNA gene of *T. pallidum* were identified by analyzing DNA extracted from PCR-positive samples. PCR, accompanied by purification using the QIAquick PCR purification kit provided by Qiagen and subsequent digestion using MboII and BsaI enzymes from New England Biolabs [41], was employed to identify mutations in rRNA genes associated with antimicrobial resistance. The primer sequences are detailed in Table 1 [15].

#### 2.5.2. Detection of the 16S rRNA G1058C mutation

Three PCR primers were employed to detect the G1058C point mutation within the 16S rRNA gene of *T. pallidum*. Table 1 provides details about these primers. After purification and sequencing, the obtained sequence was compared to the standard 16S rDNA sequence of the *T. pallidum* Nichols strain using DNA Star software [15].

### 2.6. Statistical analysis

For statistical analysis, we utilized SPSS Inc.’s Statistical Package for the Social Sciences, version 24.0 (Chicago, IL). Associations between categorical variables were identified using either the Fisher exact test or the chi-square test. Findings with a P-value below 0.05 were considered statistically significant [15].

## 3. Results

### 3.1 Screening by CMIA

In our earlier investigation [37] a total of 32,812 blood donors underwent CMIA screening for *T. pallidum*, of which 272 (0.83%) were reactive and 32,540 (99.17%) were non-reactive. In this study, a total of 32,661 blood donors (99.54%) were men, whereas 151 (0.46%) were women. In this study, 1885 donors (5.74%) gave blood voluntarily, while 30,927 (94.26%) gave blood as a replacement.

### 3.2 Screening by PCR

PCR tests were conducted on samples obtained from 272 syphilis-reactive blood donors. Out of these, 46 samples tested positive (16.91%, *P = 0*.*432*). Among these 46 PCR-positive samples, the majority exhibited positivity for all four genes. The *polA* gene was detected in all 46 samples (100%, *P<0*.*001*), the *tpp47* gene in 42 samples (91.30%, *P<0*.*001*), the *bmp* gene in 41 samples (89.13%, *P<0*.*001*), and the *tp0319* gene in 34 samples (73.91%, *P<0*.*001*) (Table 2).

**Table 2:** Different genes detected by PCR (n = 46).

Total Number of PCR Positive Samples	*PolA* Gene Detected			*tpp47* Gene Detected			*bmp* Gene Detected			*tp0319* Gene Detected		
	Count	*%*	*P Value*	Count	*%*	*P Value*	Count	*%*	*P Value*	Count	*%*	*P Value*
46	46	100	<0.001	42	91.30	<0.001	41	89.13	<0.001	34	73.91	<0.001

### 3.3 Analysis of molecular subtypes

These 46 PCR-positive samples were further subjected to molecular subtyping of *T. pallidum*, and complete subtypes were identified in 27 samples (58.69%, *P = 0*.*003*). During molecular subtyping, the number of 60-bp repeats detected in the *arp* gene was 6, 11, 12, 14, and 15; variations in the sequences of *tpr* Subfamily II genes (*tpr EGJ*) manifested as b, e, and d, along with diverse regions in the *tp0548* gene denoted as a, g, and f (Table 3). In the process of molecular subtyping, the *arp* gene exhibited 60-bp repeats with counts of 6, 11, 12, 14, and 15, identified in 1, 2, 2, 20, and 2 samples, respectively (Table 4). In the course of molecular subtyping, variations in the sequences of *tpr* subfamily II genes (*tpr* EGJ) designated as b, e, and d were detected in 2, 2, and 23 samples, respectively (Table 4). During molecular subtyping, the variable regions in the *tp0548* gene (a, g, and f) were identified in 1, 5 and 21 samples, respectively (Table 4). After combining the results of the number of 60 bp repeats in the *arp* gene, sequence variations in *tpr* subfamily II genes (*tpr* EGJ), and variable regions in the *tp0548* gene, nine different molecular subtypes were identified, including 14d/f, 14d/g, 15d/g, 12d/f, 14b/f, 12e/a, 11d/f, 14e/f, and 6d/f in 14 (51.85%), 3 (11.11%), 2 (7.40%), 1(3.70%), 2(7.40%), 1(3.70%), 2(7.40%), 1(3.70%) and 1(3.70%) of PCR-positive samples, respectively. Subtype 14d/f was the most prevalent (51.85%), while subtypes 12d/f, 12e/a, 14e/f, and 6d/f were less prevalent (3.70%) each (Table 5).

**Table 3:** Analysis of genes targeted for molecular subtyping of *T. pallidum* (n = 27).

S. NO.	Type of Analysis	Results
1.	Number of 60p Repeats in *arp* Gene	6,11,12,14,15
2.	Sequence Variations in *tpr* Subfamily II Genes (*tpr* EGJ)	b,e,d
3.	Variable Regions in *tp0548* Gene	a,g,f

**Table 4:** Sample-wise analysis of genes for molecular subtypes of *T. pallidum* (n = 27).

Number of 60bp Repeats in *arp* Gene					
Number of Repeats	6	11	12	14	15
Number of Samples	1	2	2	20	2
Sequence Variations in *tpr* Subfamily II Genes (*tpr* EGJ)					
Types of Variations	b		e	d	
Number of Samples	2		2	23	
Variable Regions in *tp0548* Gene					
Types of Variations	a		g	f	
Number of Samples	1		5	21	

**Table 5:** Complete molecular subtypes of *T. pallidum* in blood donors of Khyber Pakhtunkhwa (n = 27).

S.NO.	Molecular Subtype	Number of Positive Samples	Percentage
1.	14d/f	14	51.85%
2.	14d/g	3	11.11%
3.	15d/g	2	7.40%
4.	12d/f	1	3.70%
5.	14b/f	2	7.40%
6.	12e/a	1	3.70%
7.	11d/f	2	7.40%
8.	14e/f	1	3.70%
9.	6d/f	1	3.70%

### 3.4 Regional analysis of molecular subtypes

When these different molecular subtypes were analyzed based on their occurrence in different districts of Khyber Pakhtunkhwa, molecular subtype 14d/f was identified in districts Charsada, Mardan, Bannu, Khyber, Swat, Kohat, and Peshawar in 1, 2, 1, 1, 2, 1, and 6 blood donors, respectively. Molecular subtype 14d/g was identified in districts Kohat and Peshawar in 1 and 2 blood donors, respectively. Molecular subtype 15d/g was identified in districts Mardan and Peshawar in one blood donor each. Molecular subtype 12d/f was identified only in 1 blood donor from district Swat. Molecular subtype 14b/f was identified in districts Kohat and Peshawar in one blood donor each. Molecular sub-type 12e/a was identified only in 1 blood donor from district Swabi. Molecular subtype 11d/f was identified in districts Swabi and Peshawar in one blood donor each. Molecular sub-type 14e/f was identified only in 1 blood donor from district Peshawar. Molecular subtype 6d/f was identified only in 1 blood donor from district Dir Lower (Table 6).

**Table 6:** Regional distribution of different molecular subtypes of *T. pallidum* in blood donors of Khyber Pakhtunkhwa (n = 27).

S.NO.	District	Number of Molecular subtypes of *T. pallidum* in blood donors								
		14d/f	14d/g	15d/g	12d/f	14b/f	12e/a	11d/f	14e/f	6d/f
1.	Charsada	1	‐‐	‐‐	‐‐	‐‐	‐‐	‐‐	‐‐	‐‐
2.	Mardan	2	‐‐	1	‐‐	‐‐	‐‐	‐‐	‐‐	‐‐
3.	Bannu	1	‐‐	‐‐	‐‐	‐‐	‐‐	‐‐	‐‐	‐‐
4.	Dir Lower	‐‐	‐‐	‐‐	‐‐	‐‐	‐‐	‐‐	‐‐	1
5.	Khyber	1	‐‐	‐‐	‐‐	‐‐	‐‐	‐‐	‐‐	‐‐
6.	Swabi	‐‐	‐‐	‐‐	‐‐	‐‐	1	1	‐‐	‐‐
7.	Swat	2	‐‐	‐‐	1	‐‐	‐‐	‐‐	‐‐	‐‐
8.	Kohat	1	1	‐‐	‐‐	1	‐‐	‐‐	‐‐	‐‐
9.	Peshawar	6	2	1	‐‐	1	‐‐	1	1	‐‐

### 3.5 Analysis of point mutations linked to antibiotic resistance

These 46 samples, initially positive for PCR, underwent additional testing to detect A2058G and A2059G point mutations in 23S rRNA, along with G1058C point mutations in 16S rRNA. Among the samples, a significant presence of the A2058G point mutation in 16S rRNA was observed in 36 cases (78%, *P<0*.*001*), indicating resistance to macrolides. However, neither the A2059G mutations in 16S rRNA associated with macrolide resistance nor the G1058C mutations in 16S rRNA linked to doxycycline resistance were detected in any sample (Table 7).

**Table 7:** Analysis of point mutations in 23S r RNA and 16S r RNA of *T. pallidum* (n = 46).

S.NO	Type of Gene	Type of Mutation	Number of Samples
1.	23S rRNA	A2058G	36 (78%) P = <0.001
2.	23S rRNA	A2059G	0 (0%)
3.	16S rRNA	G1058C	0 (0%)

### 3.6 Geographical dispersion of A2058G point mutations in 23S rRNA associated with macrolide resistance

In the study, the instances of individuals exhibiting the A2058G point mutation in the 23S rRNA, which is linked to resistance against macrolide antibiotics, showed variability among various regions. Specifically, the distribution of these cases was observed as follows: Charsada: 1, Mardan: 5, Bannu: 3, Dir Lower: 1, Khyber: 1, Swabi: 2, Swat: 3, Kohat: 6, and Peshawar: 14 (Table 8).

**Table 8:** Geographical dispersion of A2058G point mutations in 23S rRNA associated with macrolide resistance (n = 36).

S.NO.	District	Number of cases having A2058G point mutation in 23S rRNA
1.	Charsada	1
2.	Mardan	5
3.	Bannu	3
4.	Dir Lower	1
5.	Khyber	1
6.	Swabi	2
7.	Swat	3
8.	Kohat	6
9.	Peshawar	14

### 3.7 Distribution of point mutations in molecular subtypes

In the present study, the distribution of macrolide resistance among different molecular subtypes, including 14d/f: 14 (36.88%), 14d/g: 3 (8.33%), 15d/g: 2 (5.55%), 12d/f: 1 (2.77%), 14b/f: 2 (5.55%), 12e/a: 1 (2.77%), 11d/f: 2 (5.55%), 14e/f: 1 (2.77%), 6d/f: 1 (2.77%), and unknown subtypes: 9 (25.00%), had varying cases of macrolide resistance (Table 9).

**Table 9:** Macrolide resistance in subtypes of *T. pallidum* (n = 36).

S.N0.	Molecular Subtype	Number of Macrolide Resistant Cases	Percentage
1.	14d/f	14	36.88
2.	14d/g	3	8.33
3.	15d/g	2	5.55
4.	12d/f	1	2.77
5.	14b/f	2	5.55
6.	12e/a	1	2.77
7.	11d/f	2	5.55
8.	14e/f	1	2.77
9.	6d/f	1	2.77
10.	Unknown Subtypes	9	25.00

## 4. Discussion

Syphilis is a transmissible infection [46]. The prevalence of syphilis has increased in Pakistan and globally; it is a widespread sexually transmitted disease (STD) both in underdeveloped and advanced nations [47–49]. To our knowledge, this is the first study in Pakistan conducted on the molecular subtyping and antibiotics resistance of *T. pallidum*. To our knowledge, globally, this is the first study on blood donors in which serum has been used exclusively on a large scale, regional molecular subtyping and detection of antimicrobial resistance were carried out to provide enhanced surveillance of syphilis. This is an effective way to assess circulating *T. pallidum* in blood donors because blood donors are healthy individuals and chancres are usually invisible; they might be overlooked, ignored, or possibly not present. Chancres samples are difficult to collect. This approach also provides an effective insight into the status of antimicrobial resistance in *T. pallidum* locally. Currently, it is possible but challenging to cultivate *T. pallidum* in vitro using a tissue culture system, and to understand this pathogen; modern molecular biology techniques provide a valuable platform.

The reactive cases of syphilis *(T. pallidum)* in the present study were 272 (0.83%). The findings in our study were higher when compared with the results of different studies conducted in some developed countries, including the USA (0.16%) [50], Italy (0.031%) [51], Israel (0.047%) [52], and Saudi Arabia (0.044%) [53]. When comparing our results with studies from various African countries, the prevalence of syphilis in blood donors was much higher than the results of the present study, for example, in Burkina Faso (1.5%) [54], Nigeria (3.1%) [55], and Angola (20.0%) [56]. Our results were slightly higher when compared with a study conducted by the Safe Blood Transfusion Programme of Pakistan, in which the prevalence of syphilis in Pakistani blood donors was 0.72% [31].

When comparing our study with earlier studies from different cities in Pakistan, the prevalence rates were Karachi (0.91%) [35], Karachi (2.1%) [34], Islamabad (1.55%) [33], Peshawar (0.43%) [57], Lahore (0.5%) [58], and Lahore (2.25%) [59]. Our result was higher (0.83%) compared to an earlier study in Peshawar, where the prevalence was 0.43%. This difference may be attributed to the time gap between these two studies; our study was conducted in 2020–2021, while the other study was conducted in 2008–2011.

To enhance the percentage of positive samples found when screening blood specimens by PCR, we amplified four separate target genes (*polA*, *tpp47*, *bmp*, and *tp0319*) by PCR in this work [18, 60]. However, our positive detection rate was lower than that recorded by other researchers. Despite the drawbacks of using blood samples, such as the presence of PCR-inhibitory substances and a low bacterial load, this approach for molecular epidemiological study is valuable. It can significantly increase the number of potential participants in subtyping evaluations, including those with asymptomatic, persistent infections.

Only 46 out of the 272 CMIA-reactive samples used in this study (16.91%) had positive PCR results. In other investigations, plasma exhibited a DNA extraction efficiency of 13.0% (range: 0.5–81.2), whereas whole blood had a 25.0% extraction efficiency (range: 13.5–41.6) [61]. In other studies, the positivity ratio of *T. pallidum* DNA varied in different sample types: blood samples had a ratio of 36.8% [62], plasma had 49% [44], serum had 36% [44], earlobe blood had 92.0% [63], serum had 66.9% [63], whole blood had 64.2% [63], blood had 72.7% [64], plasma had 18% [65], serum had 14.7% [65], whole blood had 24% [65], whole blood had 20.1% [15], whole blood had 40.3% [25], whole blood had 22.2% [27], whole blood had 42% [28], whole blood had 31.7% [66], plasma had 41.45% [66], and blood had 6.6% [67]. The lowest DNA extraction efficiency was observed in whole blood, serum, and plasma, with no discernible difference among them [61]. In three investigations, blood samples from patients with secondary syphilis yielded more DNA compared to blood from patients with primary or latent syphilis (55.8% vs. 34.1% vs. 33.6%), when the samples were subdivided by clinical stage [30, 68, 69].

In comparison to PCR, the study discovered poor levels of specificity for serological testing. The rationale is that only the specific subspecies causing venereal syphilis are detectable in our PCR-based diagnosis. Overall, our findings demonstrated the effectiveness of PCR in detecting *T. pallidum* subsp. *pallidum*, in contrast to the inability of various serological tests to distinguish between venereal syphilis and other treponematoses [70, 71]. The use of a more precise technique, such as PCR, in the detection of venereal syphilis, might aid in lowering the number of infections acquired through a blood transfusion due to the high prevalence of transfusion-transmitted infectious diseases. In addition, we found that false positives from these serological tests could result in inaccurate prevalence estimates.

Due to the difficulty of interpreting the results, the diagnosis is frequently challenging (false negatives). In Khyber Pakhtunkhwa, Pakistan, the majority of blood recipients are infants or pregnant women; hence, incorrect findings and interpretations brought on by a test’s flaw would not only harm the patient but might also result in problems or the development of congenital syphilis. Despite the drawbacks of using blood samples for molecular epidemiological research, such as the presence of PCR-inhibitory substances and low bacterial load, this approach can significantly increase the pool of potential participants in subtyping evaluations, including those with asymptomatic, persistent infection. For the subtyping of *T. pallidum* DNA from serum, we used the Enhanced Centres for Disease Control and Prevention (ECDC) typing method.

In the present study, nine distinct molecular subtypes of *T. pallidum* were found in total, including 14d/f, 14d/g, 15d/g, 12d/f, 14b/f, 12e/a, 11d/f, 14e/f, and 6d/f in 14 (51.85%), 3 (11.11%), 2 (7.40%), 1(3.70%), 2(7.40%), 1(3.70%), 2(7.40%), 1(3.70%) and 1(3.70%) of PCR-positive samples, respectively. The other studies that discovered comparable subgroups to those in our analysis include Denmark (14 d/g, 14d/f, and 14 b/f) [72], United Kingdom (14d/g and 14 d/f) [24], Argentina (14 d/f, 14d/g, and 11 d/f) [11], China (14d/f, 14e/f, 12d/f, 6d/f, 11d/f, 14b/f, 12e/a, and 14d/g) [15], Belgium (14 e/f, and 14d/g) [25], China (14d/f) [16], Italy (14d/g) [73], Denmark (14 b/f, 14 d/f, and 14d/g) [72], Peru (14d/f, 14 d/g, and 15d/g) [12], China (14d/f) [17], USA (14d/f, 14 d/g, and 15d/g) [10], China (14e/f, 12d/f, 6d/f, and 11d/f) [74], China (14d/f) [66], Taiwan (14 b/f and 14e/f) [75], France (14d/f and 14d/g) [14], USA, China, Ireland, and Madagascar (14d/f, 14 d/g, 15d/g, and 12d/f) [9], and Australia (14d/f,14d/g and 14e/f) [76].

In this study, blood donors from the majority of the districts, including Peshawar, Kohat, Swat, Khyber, Bannu, Mardan, and Charsada, were found to have subtype 14d/f, which was the most common (in 7 districts out of 9). Our results are comparable to those of other studies where the 14d/f subtype was identified as the most common, such as studies conducted in the United States and China [9], Buenos Aires, Argentina [11], Hunan, China [15], China [16], Lima, Peru [12], Shanghai, China [17], Seattle, Washington [10], and Paris, France [14].

Due to their effectiveness, relative affordability, and ease of oral administration, macrolides and doxycycline antibiotics have been frequently used to treat syphilis in non-pregnant individuals who are penicillin-allergic [77]. Earlier research revealed regional differences in macrolides resistance to *T. pallidum*, demonstrating a high incidence of resistance in several wealthy nations. The use of single-dose azithromycin became increasingly popular for treating syphilis when syphilis rates in the USA started to rise in 2000. For partner-delivered treatment of sexual encounters, this regimen proved especially effective. However, failures of azithromycin therapy were discovered in San Francisco in 2002 [78].

In the ongoing study, no mutations were detected in the rRNA gene associated with doxycycline resistance. However, 78.26% of the samples exhibited a mutation in the rRNA gene that imparts resistance to macrolides. Similarly, the other studies where resistance to macrolides was identified include the UK (66.6%) [24], Canada (28.6%) [18], Argentina (14.3%) [11], China (97.5%) [15], Belgium (100%) [25], Italy (94.3%) [73], Peru (0%) [12], USA (68%) [19], USA (53.2%) [20], Ireland (93.1%) [27], Australia (84%) [23], China (91.9%) [29], Czech Republic (66.7%) [28], Canada (16%) [21], USA (72%) [10], China (100%) [30], and the USA (64.34%) [22].

In the current study, the major cities of Khyber Pakhtunkhwa, Pakistan, including Peshawar, Kohat, and Mardan, were found to have the highest number of macrolide-resistant cases: 14, 6, and 5, respectively. In the current study, Peshawar, the largest city in Khyber Pakhtunkhwa, Pakistan, with a population comprised of individuals from all over KP, Afghanistan, and other areas of Pakistan, had the greatest number of macrolide-resistant cases. People from Afghanistan travel frequently to other nations as well as back to their own. Similarly, as previously mentioned, there appear to be geographical variations in the prevalence of macrolide-resistant *T. pallidum* strains, with the largest prevalence occurring in major towns with active tourist industries (Sydney, Shanghai, Dublin, London, San Francisco, and Seattle) [79]. In Pakistan, no such type of study had been conducted before. Studies on the frequency of macrolide-resistant *T. pallidum* in Asia are scarce [30, 45]. Even though the disease is more common and antibiotics are more frequently used in medical procedures here (and occasionally misused) [80].

In the present study, resistance to macrolides was detected in different molecular subtypes, including 14d/f, 14d/g, 15d/g, 12d/f, 14b/f, 12e/a, 11d/f, 14e/f, and 6d/f. Similar to other studies, the mutations responsible for resistance to macrolides were observed in China in Subtypes 14e/f, 12d/f, 6d/f, and 11d/f [74]; in the UK, 14d/f and 14d/g [24]; in Australia, 14b/f, 14d/f, and 14d/g [76]; in Italy, 14d/g [73]; and in the US, 14d/f and 14d/g [10].

## 5. Conclusion

We demonstrated that subtype 14d/f was the most prevalent among circulating *T. pallidum* strains in blood donors in Khyber Pakhtunkhwa, Pakistan, during the study period. Additionally, we demonstrated that the A2058G mutation, linked to macrolide resistance, was present in all these strains. However, the G1058C mutation, associated with decreased susceptibility to doxycycline, was undetected. Consequently, macrolide medications should not be used to treat syphilis in Khyber Pakhtunkhwa, Pakistan. On the other hand, further research is needed to determine whether doxycycline, a secondary antibiotic suggested by European guidelines for syphilis treatment [40], is susceptible to resistance. To adapt treatment strategies quickly to changes in the molecular epidemiology of the illness, we recommend monitoring the molecular subtypes and antibiotic resistance of successive *T. pallidum* DNA samples from syphilis patients across Pakistan.

## Supporting information

S1 File Excel sheets of syphilis data. (XLSX)

## References

[1] Tsuboi M, Evans J, Davies EP, Rowley J, Korenromp EL, Clayton T, et al. Prevalence of syphilis among men who have sex with men: a global systematic review and meta-analysis from 2000–20. The Lancet Global Health. 2021;9(8):e1110–8. doi: 10.1016/S2214-109X(21)00221-7 34246332 PMC9150735

[2] Kreisel KM, Spicknall IH, Gargano JW, Lewis FM, Lewis RM, Markowitz LE, et al. Sexually transmitted infections among US women and men: Prevalence and incidence estimates, 2018. Sexually Transmitted Diseases. 2021;48(4):208–14. doi: 10.1097/OLQ.0000000000001355 33492089 PMC10245608

[3] Table 12. Total Syphilis*—Reported Cases and Rates of Reported Cases by State/Territory and Region in Alphabetical Order, United States, 2017–2021 [Internet]. 2023 [cited 2023 Nov 6]. Available from: https://www.cdc.gov/std/statistics/2021/tables/12.htm

[4] Choudhri Y, Miller J, Sandhu J, Leon A, Aho J. Sexually transmitted infections: Infectious and congenital syphilis in Canada, 2010–2015. Canada Communicable Disease Report. 2018;44(2):43. 29770098 10.14745/ccdr.v44i02a02 PMC5864261

[5] Syphilis ‐ Annual Epidemiological Report for 2019 [Internet]. 2022 [cited 2023 Nov 6]. Available from: https://www.ecdc.europa.eu/en/publications-data/syphilis-annual-epidemiological-report-2019

[6] Chico RM, Mayaud P, Ariti C, Mabey D, Ronsmans C, Chandramohan D. Prevalence of malaria and sexually transmitted and reproductive tract infections in pregnancy in sub-Saharan Africa: a systematic review. Jama. 2012;307(19):2079–86. doi: 10.1001/jama.2012.3428 22665107

[7] Esmaeilzadeh F, Mohammadi M, Amjadipour A, Jafari A, Ghelichi-Ghojogh M, Khezri R, et al. Prevalence of Syphilis Infections among the Iranian Population: A Systematic Review and Meta-Analysis. Iranian Journal of Public Health [Internet]. 2022 Jul 17 [cited 2023 Nov 6]; Available from: https://publish.kne-publishing.com/index.php/ijph/article/view/10085 doi: 10.18502/ijph.v51i7.10085 36248307 PMC9529719

[8] Wang C, Zhao P, Xiong M, Tucker J, Ong J, Hall B, et al. New Syphilis Cases in Older Adults, 2004–2019: An Analysis of Surveillance Data From South China. Frontiers in Medicine. 2021 Dec 2;8. doi: 10.3389/fmed.2021.781759 34926524 PMC8674684

[9] Marra CM, Sahi SK, Tantalo LC, Godornes C, Reid T, Behets F, et al. Enhanced molecular typing of Treponema pallidum: geographical distribution of strain types and association with neurosyphilis. The Journal of infectious diseases. 2010;202(9):1380–8. doi: 10.1086/656533 20868271 PMC3114648

[10] Grimes M, Sahi SK, Godornes BC, Tantalo LC, Roberts N, Bostick D, et al. Two mutations associated with macrolide resistance in Treponema pallidum: increasing prevalence and correlation with molecular strain type in Seattle, Washington. Sexually transmitted diseases. 2012;39(12):954. doi: 10.1097/OLQ.0b013e31826ae7a8 23191949 PMC3668457

[11] Gallo Vaulet L, Grillová L, Mikalová L, Casco R, Rodríguez Fermepin M, Pando MA, et al. Molecular typing of Treponema pallidum isolates from Buenos Aires, Argentina: frequent Nichols-like isolates and low levels of macrolide resistance. PLoS One. 2017;12(2):e0172905. doi: 10.1371/journal.pone.0172905 28235102 PMC5325558

[12] Flores JA, Vargas SK, Leon SR, Perez DG, Ramos LB, Chow J, et al. Treponema pallidum pallidum genotypes and macrolide resistance status in syphilitic lesions among patients at 2 sexually transmitted infection clinics in Lima, Peru. Sexually transmitted diseases. 2016;43(7):465–6. doi: 10.1097/OLQ.0000000000000465 27322050

[13] Venter JME, Müller EE, Mahlangu MP, Kularatne RS. Treponema pallidum Macrolide Resistance and Molecular Epidemiology in Southern Africa, 2008 to 2018. Diekema DJ, editor. J Clin Microbiol. 2021 Sep 20;59(10):e02385–20. doi: 10.1128/JCM.02385-20 34346717 PMC8451413

[14] Grange PA, Allix-Beguec C, Chanal J, Benhaddou N, Gerhardt P, Morini JP, et al. Molecular subtyping of Treponema pallidum in Paris, France. Sexually transmitted diseases. 2013;40(8):641–4. doi: 10.1097/OLQ.0000000000000006 23859911

[15] Xiao Y, Liu S, Liu Z, Xie Y, Jiang C, Xu M, et al. Molecular subtyping and surveillance of resistance genes in Treponema pallidum DNA from patients with secondary and latent syphilis in Hunan, China. Sexually transmitted diseases. 2016;43(5):310–6. doi: 10.1097/OLQ.0000000000000445 27100768

[16] Peng RR, Yin YP, Wei WH, Wang HC, Zhu BY, Liu QZ, et al. Molecular typing of Treponema pallidum causing early syphilis in China: a cross-sectional study. Sexually transmitted diseases. 2012;39(1):42–5. doi: 10.1097/OLQ.0b013e318232697d 22183845

[17] Dai T, Li K, Lu H, Gu X, Wang Q, Zhou P. Molecular typing of Treponema pallidum: a 5-year surveillance in Shanghai, China. Journal of clinical microbiology. 2012;50(11):3674–7. doi: 10.1128/JCM.01195-12 22972832 PMC3486273

[18] Martin IE, Tsang RS, Sutherland K, Tilley P, Read R, Anderson B, et al. Molecular characterization of syphilis in patients in Canada: azithromycin resistance and detection of Treponema pallidum DNA in whole-blood samples versus ulcerative swabs. Journal of Clinical Microbiology. 2009;47(6):1668–73. doi: 10.1128/JCM.02392-08 19339468 PMC2691066

[19] Katz KA, Pillay A, Ahrens K, Kohn RP, Hermanstyne K, Bernstein KT, et al. Molecular epidemiology of syphilis—San Francisco, 2004–2007. Sexually transmitted diseases. 2010;37(10):660–3. doi: 10.1097/OLQ.0b013e3181e1a77a 20601928

[20] Workgroup AP. Prevalence of the 23S rRNA A2058G point mutation and molecular subtypes in Treponema pallidum in the United States, 2007 to 2009. Sexually transmitted diseases. 2012;794–8. doi: 10.1097/OLQ.0b013e31826f36de 23001267

[21] Martin IE, Tsang RS, Sutherland K, Anderson B, Read R, Roy C, et al. Molecular typing of Treponema pallidum strains in western Canada: predominance of 14d subtypes. Sexually transmitted diseases. 2010;37(9):544–8. doi: 10.1097/OLQ.0b013e3181d73ce1 20539263

[22] Chen CY, Chi KH, Pillay A, Nachamkin E, Su JR, Ballard RC. Detection of the A2058G and A2059G 23S rRNA gene point mutations associated with azithromycin resistance in Treponema pallidum by use of a TaqMan real-time multiplex PCR assay. Journal of Clinical Microbiology. 2013;51(3):908–13. doi: 10.1128/JCM.02770-12 23284026 PMC3592075

[23] Read P, Jeoffreys N, Tagg K, Guy RJ, Gilbert GL, Donovan B. Azithromycin-resistant syphilis-causing strains in Sydney, Australia: prevalence and risk factors. Journal of clinical microbiology. 2014;52(8):2776–81. doi: 10.1128/JCM.00301-14 24850356 PMC4136156

[24] Tipple C, McClure MO, Taylor GP. High prevalence of macrolide resistant Treponema pallidum strains in a London centre. Sexually transmitted infections. 2011;87(6):486–8. doi: 10.1136/sextrans-2011-050082 21917695

[25] Mikalová L, Grillová L, Osbak K, Strouhal M, Kenyon C, Crucitti T, et al. Molecular typing of syphilis-causing strains among human immunodeficiency virus-positive patients in Antwerp, Belgium. Sexually transmitted diseases. 2017;44(6):376–9. doi: 10.1097/OLQ.0000000000000600 28499290

[26] Giulia Ciccarese MD, Christian PS, Conte MD, Laura Colli MD, Marco Cusini MD, Stefano Ramoni MD. Enhanced Molecular Typing of Treponema pallidum subsp. pallidum strains from four Italian hospitals shows geographical differences in strain type heterogeneity, widespread resistance to macrolides, and lack of mutations associated with doxycycline resistance. Sex Transm Dis. 2018;45(4):237–42. 29465698 10.1097/OLQ.0000000000000741 PMC5847427

[27] Muldoon EG, Walsh A, Crowley B, Mulcahy F. Treponema pallidum azithromycin resistance in Dublin, Ireland. Sexually transmitted diseases. 2012;39(10):784–6. doi: 10.1097/OLQ.0b013e318269995f 23001265

[28] Grillová L, Pĕtrošová H, Mikalová L, Strnadel R, Dastychová E, Kuklová I, et al. Molecular typing of Treponema pallidum in the Czech Republic during 2011 to 2013: increased prevalence of identified genotypes and of isolates with macrolide resistance. Journal of clinical microbiology. 2014;52(10):3693–700. doi: 10.1128/JCM.01292-14 25100820 PMC4187743

[29] Chen XS, Yin YP, Wei WH, Wang HC, Peng RR, Zheng HP, et al. High prevalence of azithromycin resistance to Treponema pallidum in geographically different areas in China. Clinical Microbiology and Infection. 2013;19(10):975–9. doi: 10.1111/1469-0691.12098 23231450

[30] Martin IE, Gu W, Yang Y, Tsang RS. Macrolide resistance and molecular types of Treponema pallidum causing primary syphilis in Shanghai, China. Clinical Infectious Diseases. 2009;49(4):515–21. doi: 10.1086/600878 19583516

[31] Waheed U, e Saba N, Wazeer A, Arshad M, Zaheer HA. Epidemiology of syphilis in blood donors in Pakistan. Global Journal of Transfusion Medicine. 2020;5(1):100.

[32] Nazir S, Pracha HS, Khan A, Nazar A, Fayyaz A, Khan MS, et al. Prevalence of syphilis in Pakistani blood donors. Advancements in life sciences. 2013;1(1).

[33] Saeed M, Hussain S, Rasheed F, Ahmad M, Arif M, Rahmani MTH. Silent killers: Transfusion transmissible infections-TTI, among asymptomatic population of Pakistan. J Pak Med Assoc. 2017;67(3):369–74. 28303984

[34] Arshad A, Borhany M, Anwar N, Naseer I, Ansari R, Boota S, et al. Prevalence of transfusion transmissible infections in blood donors of Pakistan. BMC hematology. 2016;16(1):1–6. doi: 10.1186/s12878-016-0068-2 27891232 PMC5116208

[35] Sultan S, Murad S, Irfan SM, Biag MA. Trends of venereal infections among healthy blood donors at Karachi. Archives of Iranian medicine. 2016;19(3):0–0. 26923891

[36] Saba N, Nasir JA, Waheed U, Aslam S, Mohammad I, Wazeer A, et al. Seroprevalence of transfusion-transmitted infections among voluntary and replacement blood donors at the Peshawar Regional Blood Centre, Khyber Pakhtunkhwa, Pakistan. Journal of Laboratory Physicians. 2021;13(02):162–8. doi: 10.1055/s-0041-1729485 34483564 PMC8409124

[37] Saba N, Muhammad I, Waheed U, Latif Z, Ullah W, Muhammad N, et al. Seroepidemiology and Risk Factors Analysis of Syphilis: A Cross-Sectional Study from Peshawar, Pakistan. Journal of The Society of Obstetricians and Gynaecologists of Pakistan. 2022;12(1):68–72.

[38] Pillay A, Liu H, Chen CY, Holloway B, Sturm AW, Steiner B, et al. Molecular subtyping of Treponema pallidum subspecies pallidum. Sexually transmitted diseases. 1998;408–14. doi: 10.1097/00007435-199809000-00004 9773432

[39] Tipple C, Hanna MO, Hill S, Daniel J, Goldmeier D, McClure MO, et al. Getting the measure of syphilis: qPCR to better understand early infection. Sexually transmitted infections. 2011;87(6):479–85. doi: 10.1136/sti.2011.049494 21752804 PMC3252622

[40] Janier áM, Hegyi V, Dupin N, Unemo M, Tiplica GS, Potočnik M, et al. 2014 European guideline on the management of syphilis. Journal of the European Academy of Dermatology and Venereology. 2014;28(12):1581–93. doi: 10.1111/jdv.12734 25348878

[41] Lukehart SA, Godornes C, Molini BJ, Sonnett P, Hopkins S, Mulcahy F, et al. Macrolide resistance in Treponema pallidum in the United States and Ireland. New England Journal of Medicine. 2004;351(2):154–8. doi: 10.1056/NEJMoa040216 15247355

[42] Matějková P, Flasarova M, Zakoucka H, Bořek M, Křemenová S, Arenberger P, et al. Macrolide treatment failure in a case of secondary syphilis: a novel A2059G mutation in the 23S rRNA gene of Treponema pallidum subsp. pallidum. Journal of medical microbiology. 2009;58(6):832–6. 19429763 10.1099/jmm.0.007542-0

[43] Attie A, de Almeida-Neto C, S Witkin S, Derriga J, Nishiya AS, Ferreira JE, et al. Detection and analysis of blood donors seropositive for syphilis. Transfusion Medicine. 2021;31(2):121–8. doi: 10.1111/tme.12761 33480044

[44] Liu H, Rodes B, Chen CY, Steiner B. New tests for syphilis: rational design of a PCR method for detection of Treponema pallidum in clinical specimens using unique regions of the DNA polymerase I gene. Journal of Clinical Microbiology. 2001;39(5):1941–6. doi: 10.1128/JCM.39.5.1941-1946.2001 11326018 PMC88053

[45] Wu H, Chang SY, Lee NY, Huang WC, Wu BR, Yang CJ, et al. Evaluation of macrolide resistance and enhanced molecular typing of Treponema pallidum in patients with syphilis in Taiwan: a prospective multicenter study. Journal of clinical microbiology. 2012;50(7):2299–304. doi: 10.1128/JCM.00341-12 22518868 PMC3405618

[46] Carlos, Avelleira R, Bottino G. Syphilis: diagnosis, treatment and control Sífilis: diagnóstico, tratamento e controle. An Bras Dermatol. 2006;81(2):111–26.

[47] Allain JP, Stramer SL, Carneiro-Proietti ABF, Martins ML, da Silva SNL, Ribeiro M, et al. Transfusion-transmitted infectious diseases. Biologicals. 37(2):71–7. doi: 10.1016/j.biologicals.2009.01.002 19231236

[48] Waheed U, Khan H, Satti HS, Ansari MA, Malik MA, Zaheer HA. Prevalence of Transfusion Transmitted Infections among Blood Donors of a Teaching Hospital in Islamabad.:4.

[49] Mahmood H, Ansari US, Aslam M. Prevalence of syphilis and HIV infection in blood donors in cosmopolitan Lahore during the year 2014. Journal of University Medical & Dental College. 2015;6(4):42–5.

[50] Kane MA, Bloch EM, Bruhn R, Kaidarova Z, Murphy EL. Demographic determinants of syphilis seroprevalence among U.S. blood donors, 2011–2012. BMC Infectious Diseases. 2015 Feb 15;15(1):63. doi: 10.1186/s12879-015-0805-3 25887811 PMC4369345

[51] Drago F, Cogorno L, Ciccarese G, Strada P, Tognoni M, Rebora A, et al. Prevalence of syphilis among voluntary blood donors in Liguria region (Italy) from 2009 to 2013. International Journal of Infectious Diseases. 2014;28:45–6. doi: 10.1016/j.ijid.2014.04.008 25200092

[52] Vera L, Milka D, Nurith SL, Eilat S. Prevalence and Incidence of Syphilis among Volunteer Blood Donors in Israel. Journal of Blood Transfusion. 2014 Apr 22;2014:e154048. doi: 10.1155/2014/154048 24860686 PMC4016887

[53] Elyamany G, Al Amro M, Pereira WC, Alsuhaibani O. Prevalence of Syphilis among Blood and Stem Cell Donors in Saudi Arabia: An Institutional Experience. Electron Physician. 2016 Aug;8(8):2747–51. doi: 10.19082/2747 27757184 PMC5053455

[54] Bisseye C, Sanou M, Nagalo BM, Kiba A, Compaoré TR, Tao I, et al. Epidemiology of syphilis in regional blood transfusion centres in Burkina Faso, West Africa. Pan African Medical Journal. 2014;16(1). 10.11604/pamj.2013.16.69.2767 PMC3976651 24711869

[55] Okoroiwu HU, Okafor IM, Asemota EA, Okpokam DC. Seroprevalence of transfusion-transmissible infections (HBV, HCV, syphilis and HIV) among prospective blood donors in a tertiary health care facility in Calabar, Nigeria; an eleven years evaluation. BMC public health. 2018;18(1):1–8. 10.1186/s12889-018-5555-x PMC5964952 29788937

[56] Quintas E, Cogle ADC, Dias CC, Sebastiao A, Pereira ADC, Sarmento A, et al. Prevalence of Syphilis in Blood Donors in Angola from 2011 to 2016. Clinical and Medical Reports. 2018;1(4).

[57] Attaullah S, Khan S, Khan J. Trend of transfusion transmitted infections frequency in blood donors: provide a road map for its prevention and control. Journal of translational medicine. 2012;10(1):1–5. doi: 10.1186/1479-5876-10-20 22293125 PMC3286364

[58] Manzoor I, Hashmi N, Daud S, Ajmal S, Fatima H, Rasheed Z, et al. Seroprevalence of transfusion transmissible infections (TTIS) in blood donors. Biomedica. 2009;25(10):154–8.

[59] Zahoor S, Hameed S, Iqbal W, ur Rehman H, ur Rehman N, Jaral J. Seroprevalence of Syphilis in healthy blood donors of Lahore during year 2016 and 2017; An upcoming problem for Pakistan. The Professional Medical Journal. 2020;27(01):138–42.

[60] Woznicová V, Šmajs D, Wechsler D, Matejková P, Flasarová M. Detection of Treponema pallidum subsp. pallidum from skin lesions, serum, and cerebrospinal fluid in an infant with congenital syphilis after clindamycin treatment of the mother during pregnancy. Journal of Clinical Microbiology. 2007;45(2):659–61. doi: 10.1128/JCM.02209-06 17151205 PMC1829021

[61] Peng RR, Wang AL, Li J, Tucker JD, Yin YP, Chen XS. Molecular typing of Treponema pallidum: a systematic review and meta-analysis. PLoS neglected tropical diseases. 2011;5(11):e1273. doi: 10.1371/journal.pntd.0001273 22087340 PMC3210736

[62] Sutton MY, Liu H, Steiner B, Pillay A, Mickey T, Finelli L, et al. Molecular subtyping of Treponema pallidum in an Arizona County with increasing syphilis morbidity: use of specimens from ulcers and blood. The Journal of infectious diseases. 2001;183(11):1601–6. doi: 10.1086/320698 11343208

[63] Zhang Y, Dai X, Ren Z, Lin H, Cao W, Ye X. A novel nested real-time polymerase chain reaction for Treponema pallidum DNA in syphilis biospecimens. Sexually transmitted diseases. 2019;46(1):41–6. doi: 10.1097/OLQ.0000000000000908 30247262

[64] Casal CAD, Silva MO da, Costa IB, Araújo E da C, Corvelo TC de O. Molecular detection of Treponema pallidum sp. pallidum in blood samples of VDRL-seroreactive women with lethal pregnancy outcomes: a retrospective observational study in northern Brazil. Revista da Sociedade Brasileira de Medicina Tropical. 2011;44:451–6. doi: 10.1590/s0037-86822011005000047 21789353

[65] Grange PA, Gressier L, Dion PL, Farhi D, Benhaddou N, Gerhardt P, et al. Evaluation of a PCR test for detection of Treponema pallidum in swabs and blood. Journal of clinical microbiology. 2012;50(3):546–52. doi: 10.1128/JCM.00702-11 22219306 PMC3295187

[66] Lu Y, Wu Q, Wang L, Ji L. Molecular epidemiological survey of Treponema pallidum in pregnant women in the Zhabei District of Shanghai. Journal of medical microbiology. 2017;66(4):391–6. doi: 10.1099/jmm.0.000441 28126049

[67] Simpore A, Bisseye C, Nagalo BM, Sanou M, Nébié Y, Ghoma-Linguissi LS, et al. Importance of extending the use of polymerase chain reaction in the diagnosis of venereal syphilis in a blood transfusion center in Burkina Faso, West Africa. The Pan African Medical Journal. 2014;18. 10.11604/pamj.2014.18.56.3850 PMC4473781 26113890

[68] Castro R, Prieto E, Aguas MJ, Manata MJ, Botas J, Martins Pereira F. Molecular subtyping of Treponema pallidum subsp. pallidum in Lisbon, Portugal. Journal of clinical microbiology. 2009;47(8):2510–2. doi: 10.1128/JCM.00287-08 19494073 PMC2725690

[69] Cruz AR, Pillay A, Zuluaga AV, Ramirez LG, Duque JE, Aristizabal GE, et al. Secondary syphilis in Cali, Colombia: new concepts in disease pathogenesis. PLoS neglected tropical diseases. 2010;4(5):e690. doi: 10.1371/journal.pntd.0000690 20502522 PMC2872645

[70] Morand JJ, Simon F, Garnotel E, Mahé A, Clity E, Morlain B. Overview of endemic treponematoses. Medecine tropicale: revue du Corps de sante colonial. 2006;66(1):15–20. 16615610

[71] Farhi D, Dupin N. Diagnostic sérologique de la syphilis. In: Annales de dermatologie et de vénéréologie. Elsevier; 2008. p. 418–25. 10.1016/j.annder.2008.02.007 18457735

[72] Salado-Rasmussen K, Cowan S, Gerstoft J, Kiellberg Larsen H, Hoffmann S, Bygum Knudsen T, et al. Molecular typing of Treponema pallidum in Denmark: a nationwide study of syphilis. Acta dermato-venereologica. 2016;96(2):202–6. doi: 10.2340/00015555-2190 26122912

[73] Giacani L, Ciccarese G, Puga-Salazar C, Dal Conte I, Colli L, Cusini M, et al. Enhanced Molecular Typing of Treponema pallidum subspecies pallidum Strains From 4 Italian Hospitals Shows Geographical Differences in Strain Type Heterogeneity, Widespread Resistance to Macrolides, and Lack of Mutations Associated With Doxycycline Resistance. Sex Transm Dis. 2018 Apr;45(4):237–42. doi: 10.1097/OLQ.0000000000000741 29465698 PMC5847427

[74] Li Y, Li J, Hu W, Luo H, Zhou J, Li C, et al. Gene subtype analysis of Treponema pallidum for drug resistance to azithromycin. Experimental and therapeutic medicine. 2018;16(2):1009–13. doi: 10.3892/etm.2018.6241 30116352 PMC6090287

[75] Wu BR, Yang CJ, Tsai MS, Lee KY, Lee NY, Huang WC, et al. Multicentre surveillance of prevalence of the 23S rRNA A2058G and A2059G point mutations and molecular subtypes of Treponema pallidum in Taiwan, 2009–2013. Clinical Microbiology and Infection. 2014;20(8):802–7. doi: 10.1111/1469-0691.12529 24438059

[76] Read P, Tagg KA, Jeoffreys N, Guy RJ, Gilbert GL, Donovan B. Treponema pallidum strain types and association with macrolide resistance in Sydney, Australia: new TP0548 gene types identified. Journal of clinical microbiology. 2016;54(8):2172–4. doi: 10.1128/JCM.00959-16 27194693 PMC4963524

[77] Marra CM, Colina AP, Godornes C, Tantalo LC, Puray M, Centurion-Lara A, et al. Antibiotic selection may contribute to increases in macrolide-resistant Treponema pallidum. The Journal of infectious diseases. 2006;194(12):1771–3. doi: 10.1086/509512 17109351

[78] Chen JC. Update on emerging infections: news from the Centers for Disease Control and Prevention. Brief report: azithromycin treatment failures in syphilis infections–San Francisco, California, 2002–2003. Annals of emergency medicine. 2004;44(3):232–4. doi: 10.1016/S0196064404005748 15332064

[79] Šmajs D, Paštěková L, Grillová L. Macrolide resistance in the syphilis spirochete, Treponema pallidum ssp. pallidum: can we also expect macrolide-resistant yaws strains? The American journal of tropical medicine and hygiene. 2015;93(4):678. doi: 10.4269/ajtmh.15-0316 26217043 PMC4596581

[80] Radyowijati A, Haak H. Improving antibiotic use in low-income countries: an overview of evidence on determinants. Social science & medicine. 2003;57(4):733–44. doi: 10.1016/s0277-9536(02)00422-7 12821020

